# Direct-Write Bioprinting Approach to Construct Multilayer Cellular Tissues

**DOI:** 10.3389/fbioe.2019.00478

**Published:** 2020-01-21

**Authors:** Elahe Masaeli, Christophe Marquette

**Affiliations:** ^1^Department of Cellular Biotechnology, Cell Science Research Center, Royan Institute for Biotechnology, ACECR, Isfahan, Iran; ^2^3d.FAB, Univ Lyon, Université Lyon1, CNRS, INSA, CPE-Lyon, ICBMS, UMR 5246, Bat. Lederer, Villeurbanne, France

**Keywords:** tissue regeneration, cell layer, inkjet bioprinting, living biopaper, tissue complexity

## Abstract

As a cellular-assembly technique, bioprinting has been extensively used in tissue engineering and regenerative medicine to construct hydrogel-based three-dimensional (3D) tissue-like models with prescribed geometry. Here, we introduced a unique direct-write bioprinting strategy to fabricate a bilayer flat tissue in a hydrogel-free approach. A printed retina pigmented epithelium layer (RPE) was applied as living biopaper for positioning a fibroblast layer without using any hydrogel in bioink. We adjusted the number of cells in the inkjet droplets in order to obtain a uniform printed cell layer and demonstrated the formation of a bilayer construct through confocal imaging. Since our printing system introduced low levels of shear stress to the cells, it did not have a negative effect on cell survival, although cell viability was generally lower than that of control group over 1 week post-printing. In conclusion, our novel direct-write bioprinting approach to spatiotemporally position different cellular layers may represent an efficient tool to develop living constructs especially for regeneration of complex flat tissues.

## Introduction

Current regenerative medicine systems aim to develop three dimensional (3D) engineered constructs mimicking, as much as possible, the natural tissues found in human body. In this regard, different biofabrication approaches, based on lithography, liquid extrusion and mechanical deposition, were developed to precisely create 3D tissue scaffolds with controlled composition, microarchitecture and geometry (Mota et al., 2015; Moroni et al., 2018).

Most of the aforementioned techniques rely on the active role of biomaterials as structural units and do not incorporate cells during the manufacturing process. Consequently, following the fabrication process, a subsequent cell seeding procedure on/within scaffolds is required. In particular, due to the high complexity of living tissues, multiple cells shall be seeded in the same scaffold, using various complicated seeding methods (perfusion, diffusion, rotational seeding) (Van Den Dolder et al., 2003; Nieponice et al., 2008). In some complex layered structures, such as skin, cornea, retina, and trachea, achieving an effective cellular seeding of the architecture is a considerable challenge. Another challenging group of tissue is the osteochondral joint, where the seeding is even more difficult because of the complexity of the interfaces between cellular layers (Atala et al., 2012). To address these challenges, several methods, such as microencapsulation (Orive et al., 2015), loading cells into microfibers/beads (Matsunaga et al., 2011; Onoe and Takeuchi, 2015) and preparation of cell-laden hydrogels are currently under study to incorporate cells into the structure and use these materials as building blocks in top-down tissue engineering approaches.

Bioprinting, as a well-known additive manufacturing process, aimed at the direct construction of cell-laden tissues by utilizing one of the above-mentioned biofabrication approaches. Bioprinting is capable of either patterning materials in a well-defined design and localizing biological components, such as living cells with a controlled geometry to meet clinical needs (Gao et al., 2016; Mandrycky et al., 2016). In doing so, one of the main limitations of typical tissue engineering methods, i.e., control of the cells seeding within scaffold for mimicking complex tissues, can be overcame.

There are two major bioprinting approaches in literatures: hydrogel-based and hydrogel-free bioprinting. In the first approach, cells are printed within a hydrogel network as a supportive matrix for cell proliferation and maturation, while in the second one, cell suspensions are directly used as bioink. This hydrogel-free approach allows cells to aggregate and secrete their own extracellular matrix (ECM) to hold them together (Jakab et al., 2008; Ozbolat, 2015). Nevertheless, since proliferation is low in these scaffold-free systems, starting with a high cell population is mandatory to reduce time of tissue maturation (Ozbolat, 2015).

Usually, in hydrogel-free systems, cell aggregates or tissue spheroids are accurately positioned through one of most common bioprinting approaches, such as inkjet (Daly and Kelly, 2019), laser guidance (Barron et al., 2004), and extrusion bioprinting (Norotte et al., 2009; Jakab et al., 2010; Pourchet et al., 2017). In the meantime, there is some efforts to directly deposit cell suspension into pre-define patterns (Xu et al., 2005; Calvert, 2007), but to the best of our knowledge, controlling layer by layer the position as well as density and uniformity of seeded cells into 3D structures was not yet optimized. The purpose of this study is to facilitate and control the transfer of two different cell types in a layered structure. An inkjet bioprinting system was applied to directly print cells without carrier material in a pre-defined design. Gelatin methacryloyl (GelMa) hydrogel coated cover glass slides were used as cell-adhesive culture substrate, also called “biopaper” on which cells were bioprinted. Using this method we were able to produce complex multilayer cellular models especially useful for soft tissue engineering.

## Materials and Methods

### Printing System

The inkjet bioprinting system has been applied in our earlier study and is briefly summarized here (Masaeli et al., 2019). This is a piezoelectric inkjet dispenser S3 sciFLEXARRAYER (Scienion AG, Germany) composed of two key subsystems: a 3D stage movement system controlled by a stage controller (accuracy 5 μm) and a droplet deposition system controlled by a pulse generator and equipped with an 80 μm diameter glass nozzle, used here as a non-contact print head. The cell droplet generation system is employed to generate building blocks (i.e., cells in culture media with volume of 300 pL), guided by a computer-aided design software. A stroboscopic camera allows visual monitoring to adjust piezo voltages and pulse durations for reliable droplet ejection. This system provides precise spatial control over droplet deposition. The obtained accuracy of the droplet positioning is 5 μm.

Since, the height of printer glass nozzle is less than depth of tissue culture plates, we designed and 3D printed a silicon cylinder part, insert in each well in order to reduce the distance between the printing head and the cover glass (drop distance) to an optimum of 1 mm (**Figure 2A**). This leads the drops to be accurately deposited and not sprayed over the cover glass surface.

### Preparation of GelMa Coated Slides

A thin layer of GelMa was coated on circular glass coverslips (0.5 mm thick, 17 mm diameter, T&Q, China), and subsequently used as biopaper substrate for bioprinting. To prepare this GelMa layer, the following protocol was implemented. Irgacure D-2959 (Sigma, France), used as a photoinitiator, was dissolved in dimethyl sulfoxide (DMSO) at a concentration of 1.5% (w/v). Then, coating solution was prepared by dissolving GelMa powder (Sigma, France) at a concentration of 0.2% w/v in the previously prepared Irgacure solution, degassing under vacuum for 10 min to eliminate bubbles and finally heating for 30 min at 55°C. After setting the glass substrate on the spin coater vacuum holder, 200 μL of GelMa coating solution was dropped and spin-coated at 3,000 rev. min^−1^ for 30 s using a WS-650MZ spin coater (Laurell, USA). Finally, in order to obtain fully cross-linked coating, the spin coated slides were exposed to 32 W UV radiation (254 nm) at a distance of 14 cm for 30 min (BLX-E254, Bio-Link, Fisher Biotec, Australia).

### Cells' Printing

Green fluorescent protein (GFP)-expressing fibroblasts (NIH3T3/GFP, AKR-214, Cell Biolabs Inc., US) were expanded and suspended in D-MEM (high glucose) (Gibco, France) supplemented with 10% (v/v) fetal bovine serum (FBS, Gibco, France), 0.1 mM MEM non-essential amino acids (NEAA, Invitrogen, France), 2 mM L-glutamine (Gibco, France) and 1% (w/v) penicillin/streptomycin (10,000 U/mL) (Gibco, France).

Retina pigmented epithelium (RPE) cells (ARPE-19, ATCC, France) were expanded and suspended in DMEM-F12 medium (ATCC, France) supplemented with 10% (v/v) fetal bovine serum (FBS, Gibco, France), and 1% (v/v) penicillin/streptomycin (10,000 U/mL) (Gibco, France).

All cells were culture in a 37°C incubator in the presence of 5% CO_2_.

Just before bioprinting, cells were trypsinized [0.25% (v/v) trypsin-EDTA (ThermoFisher, France)], and counted. Cell suspensions (2.3 × 10^7^ and 6.4 × 10^7^ cells/mL for NIH3T3 and RPE, respectively) were directly used as bioink within the inkjet process using a direct-write bioprinting strategy. RPE cells were bioprinted first (18 × 18 spots array, 800 μm spot-to-spot distance, 15 nL per spot) on GelMa coated slide, adhered and cultured for 1 week. Then, NIH3T3 cells were bioprinted onto mature RPE layer following a predefined pattern. In this step, a dot pattern of 160 deposition locations of 15 nL drops were dispensed within an array of 18^*^18 spots (800 μm spot-to-spot distance) (**Figure 2B**). Images of the printed dots were recorded with an Olympus IX51 microscope.

### Cell Viability

The post-printing cellular viability was assessed using the TOX8 Resazurin-based *in vitro* Toxicology Assay Kit (Sigma, France), on media collected 1, 3, and 5 days after bioprinting, according to the manufacturer's instructions. In Brief, kit mix, 10% (v/v) of the final volume, was added to each sample and incubated during 2 h at 37°C. Resazurin (non-fluorescent) to resorufin (fluorescent) conversion was measured fluorometrically (Ex/Em = 600/690 nm) using an Infinite M200 Microplate reader (TECAN, France). Manually seeded cells with similar density were used as control group. The assays were performed three times in all experiments to assess variability.

### Actin Cytoskeleton Staining

In order to visualize F-actin structures within cells after bioprinting, constructs were fixed with 3.7% (v/v) paraformaldehyde diluted in phosphate buffer saline (PBS, Invitrogen, France) for 30 min at room temperature, permeabilized with 0.1% (v/v) TritonX-100 for 10 min, and finally stained with 5 units of Alexa Fluor 546 phalloidin (Molecular Probes, France) for 40 min at room temperature. Samples were counterstained with the nuclear stain, 4′,6-diamidino-2-phenylindole (DAPI, Invitrogen, France) (0.1 mg/mL) and imaged by confocal microscopy. Images were taken at the Center Technologique des microstructures (University of Lyon, France) using a Zeiss LSM800 confocal microscope.

### Statistical Analysis

Statistical analysis was carried out using one-way analysis of variance (ANOVA) and independent sample *t-*test to compare the viability of bioprinted cell and control group. A value of *p* ≤ 0.05 was considered statistically significant.

## Results and Discussion

The classical bioprinting strategy to create multicellular tissue models is based on designed deposition of different cell sources within a hydrogel (i.e., cell-laden hydrogel). These models normally lack uniformity of printed cells and thereupon cannot completely mimic tissue structure. Furthermore, depending on the carrier hydrogel viscosity, cells might experience high amounts of shear stress that may unfavorably affects viability, signaling and generate phenotype drifting (Blaeser et al., 2016; Chimene et al., 2016). Direct printing of living cells without hydrogel inks has then here a number of obvious advantages, such as high cell viability but also the fact that in the absence of carrier, cells will freely produce their own extracellular matrix (ECM) and form 3D structures recapitulating physiological tissues' organization (Ozbolat, 2015).

**Figure 1:**
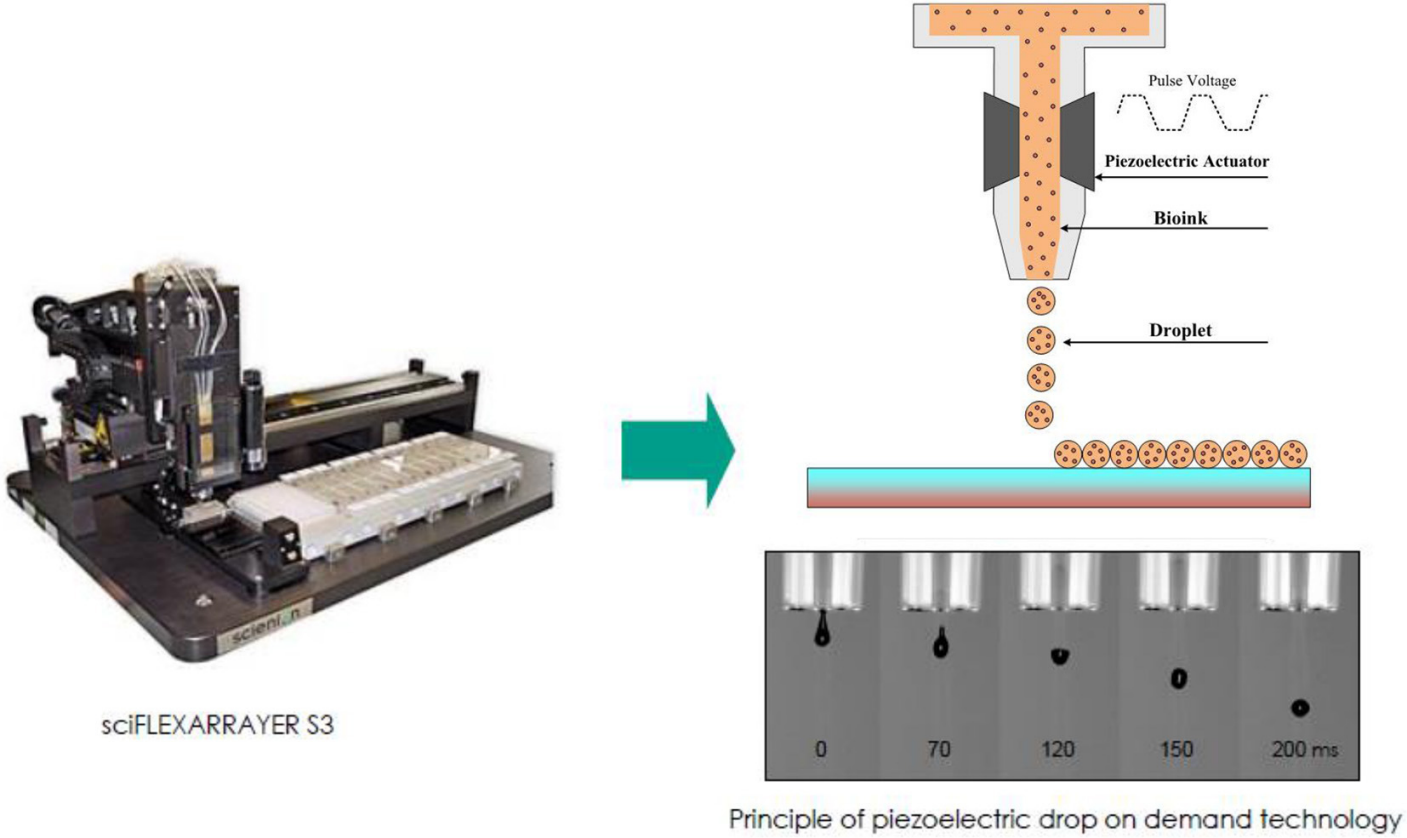
A schematic illustration of direct-write bioprinter setup (www.scienion.de).

Based on this idea, we applied a direct-write bioprinting setup to reproduce a bilayer construct in a hydrogel-free manner (Figure 1). The technique is based on a programmable non-contact piezoelectric inkjet bioprinter with a resolution of 5 μm and a minimum deposition volume of 300 pL. Such a system has been frequently applied in researches, especially for ultra-low volume liquid handling of nanoparticles (Scherbahn et al., 2016), drugs (Tronser et al., 2018), and biomolecules, such as proteins (Kilb et al., 2019) and antibodies (Marquette et al., 2012; Schulz et al., 2019).

In this bioprinting process, a crucial components, named biopaper, acts as a biomimetic tissue fusion-permissive substrate with appropriate biocompatibility and mechanical stability. In different studies, gelatin-derived hydrogels (Imani et al., 2011; Pirlo et al., 2012; Colosi et al., 2016) as well as cell-laden bioinks (Nichol et al., 2010; Bertassoni et al., 2014) have been applied as biopaper. In the present study, a 5–20 μm thick GelMa layer [measured using confocal 3D optical profilometer (NanoJura, France)] was coated on glass slide and used as biopaper to enhance the adhesion of the first layer of printed cells. GelMa is a photopolymerizable material composed of modified natural ECM components, containing then significant amount of matrix metalloproteinase and focal adhesion sequences, beneficial to promote cellular functions (Yue et al., 2015). We also previously showed that printed cells were viable and proliferate on GelMa layer over 1-week culture time, and that GelMa coating probably guides cells to form tight junction monolayer sheet (Masaeli et al., 2019).

**Figure 2:**
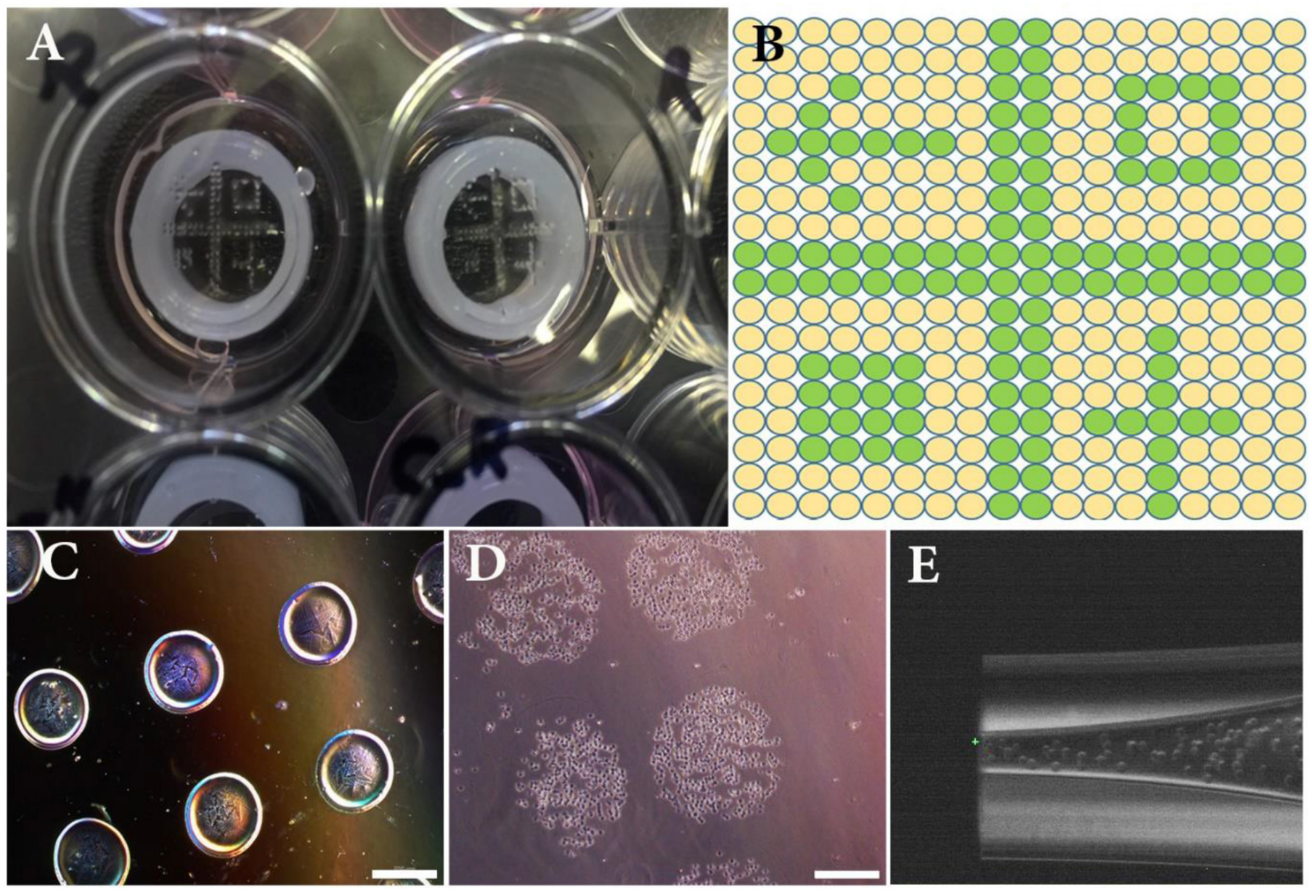
**(A)** Overview of the experimental set-up composed of a 12-well plate, 3D printed silicone positioning systems, GelMa coated circular glass slides and printed patterns of NIH3T3 cells. **(B)** Overview of the theoretical printing patterns in printer software. **(C)** Printed drops (15 nL) of alginate hydrogel used to setup size and distance between droplets. **(D)** Printed drops (15 nL) of NIH3T3 immediately after bioprinting. **(E)** Close-up image of the bioprinted nozzle filled with NIH3T3 cells. Scale bars: 500 μm.

The cell-printing system was first characterized with NIH3T3 cell suspension using the GelMa coated glass slide as printing support. Figures 2A,B depict the dot pattern design which was used to experimentally evaluate size and distance between printed dots (i.e., printing resolution). For sake of comparison, a low viscosity alginate solution was used in addition to the cell suspension. Analysis of captured images showed that average dot diameters were 426 ± 15 and 629 ± 10 μm for alginate and cell solutions, respectively (Figures 2C,D). Average drop-to-drop distances were found to be 376 ± 34 and 210 ± 12 μm for alginate and printed solutions, respectively. These measurements helped us determine the final resolution of our inkjet bioprinting system. Indeed, it is well-known that the final resolution of an inkjet printing process can be quite different from the theoretical resolution since the surface chemistry and the ink composition both lead to droplet spreading variations (Binder et al., 2011). As a consequence here, even if the printer should be able to reliably print with resolution of 5 μm, the obtained depositions expanded by 47% between alginate and cell solution and their distance therefore decreased by 44%.

Although some researchers believe that low cell density of inkjet bioprinting is one of its main disadvantages (Holzl et al., 2016; Derakhshanfar et al., 2018), here we were able to use high cell population in each drop (2.3 × 10^7^ cells/mL), probably thanks to the low viscosity (1.00E^−03^ Pa·s) of the bioink cell suspension. Especially for creation of some tissues, such as endothelium, printing with high initial cell densities is essential because cells should be in physical contact with each other. Also, high density of cells is required for tissue engineering when cells with limited to no proliferative potential (such as photoreceptors and chondrocytes…) are introduced into bioprinter (Guillotin et al., 2010).

Another concern related to bioink rheological behavior is that inkjet nozzle could be clogged or generating too much shear stress during cell deposition (Zhang and Zhang, 2015; Chimene et al., 2016; Bishop et al., 2017). Attempts have been made to overcome these issues by using low viscosity bioinks. For example, Desimone et al. (2015) reduced shear stress inside nozzle by using low viscosity solutions of recombinant spider silk instead of native silk. Colosi et al. prepared a blend of GelMa and alginate as low viscosity (0.08 Pa/s) ink and used it for printing heterogeneous 3D tissue constructs. They believed that printing with low viscosity bioinks shall enhance biological properties and resulting in tissue functions recapitulation (Colosi et al., 2016).

In the present work, not only no clogging was evidenced within the capillary nozzle (Figure 2E), but also, the calculated maximum shear stress [calculated using the nozzle geometry and a viscosity of 1.00E^−03^ Pa·s: Wall Shear rate: 1.31E^+08^ s^−1^; Wall Shear stress: 1.31E^+05^ Pa (3d.FAB, 2018)] was much lower than the previously reported acceptable stress limit (Malda et al., 2013).

**Figure 3:**
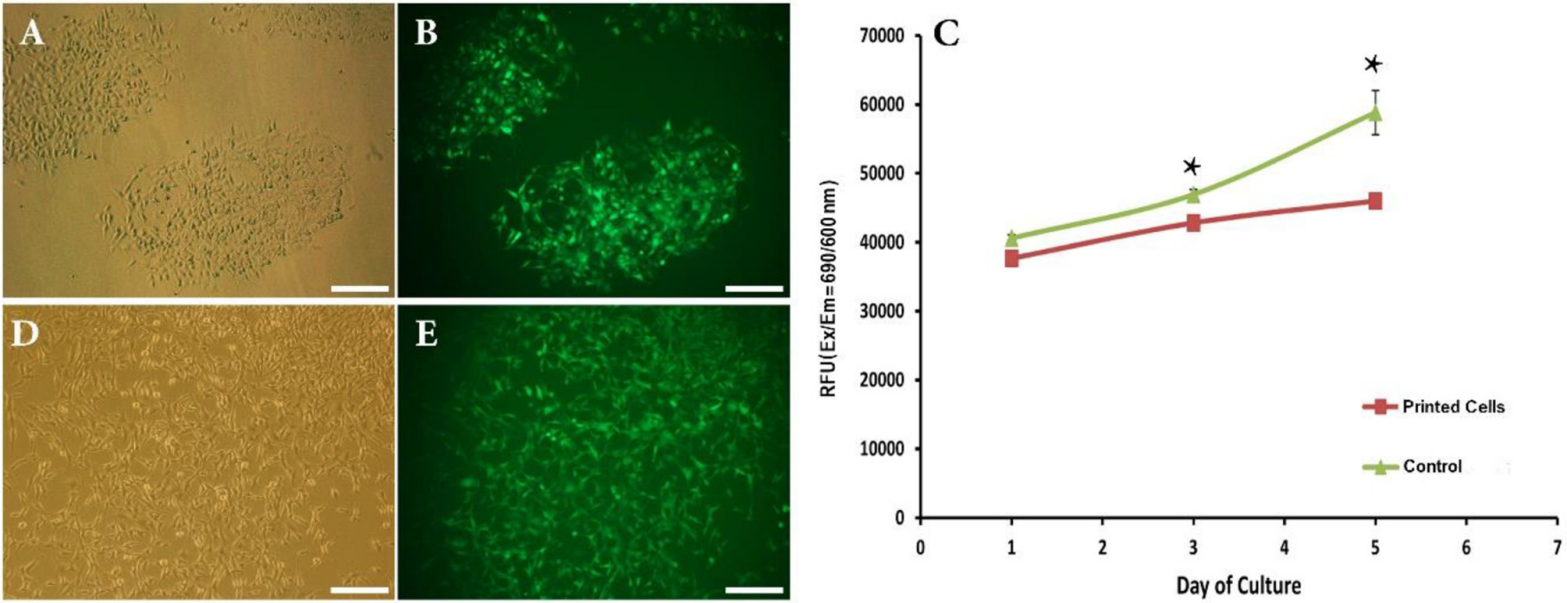
**(A,B)** Phase contrast and fluorescent images showing attachment of bioprinted NIH3T3 cells 1 day after printing. **(C)** Viability of bioprinted NIH3T3 cells during 5 days after printing. **(D,E)** Phase contrast and fluorescent images showing growth of bioprinted NIH3T3 cells 5 days after printing. Asterisks represent significant difference at *p* ≤ 0.05. Scale bars: 200 μm.

We have thus successfully replaced bioprinting ink with cell suspension and set size and distances between inkjet droplets. The next crucial step toward the introduction of this direct-write bioprinting approach is the demonstration of attachment and growth of bioprinted cells over time. This was performed by following cell behavior for 5 days post-bioprinting. Figures 3A,B depict images of two adjacent depositions using contrast phase and fluorescent microscopy, respectively. As a matter of fact, bioprinted cells had survived the inkjet process (GFP production is here a clear indicator 1 day after printing).

This qualitative analysis was reinforced by a quantitative study of the cell viability using the TOX8 Resazurin-based *in vitro* assay. Figure 3C depicts the obtained results. As can be seen, although the present inkjet bioprinting process induced significant long-term alterations in the proliferation potential of cells (days 3 and 5) when compared to control cells, our hydrogel-free system does not significantly affect cell viability immediately after printing (day 1). In similar way, Blaeser et al. (2016) have stated that printing-induced shear stress does not only have an immediate impact on cell viability but also on their long term fate. Moreover, as shown in Figures 3D,E, a clear cell proliferation can be observed, 5 days after bioprinting, evidenced by a colonization of the inter-deposition space by the growing cells, leading to a quasi-confluent cell layer.

In order to fully demonstrate the potential of the developed technique, a 2-layer cell assembly was studied. Here, we applied our hydrogel-free bioprinting approach to directly and uniformly write two cellular layers on top of each other. The final goal being to overcome, through the use of inkjet deposition, the cell seeding variability issues usually experienced using traditional co-culture strategies for layer-by-layer deposition, particularly for population of sensitive cells (Reynolds et al., 2018).

To do so, we have first bioprinted a homogeneous layer of retina pigmented epithelium (RPE) on a GelMa biopaper and cultured them for 7 days (229 ± 19 μm spot distance, 15 nL per spot). Then, once the RPE layer dense enough to cover all the biopaper surface, a second layer of NIH3T3 was printed following a tight deposition pattern (210 ± 12 μm spot distance, 15 nL per spot), chosen to lead to a homogeneous second layer.

RPE cells were selected due to their ability to form cellular monolayer sheet (epithelium layer), a very good substrate for subsequent orientated cell attachment. Numerous studies have been heretofore carried out to model complex flat tissues by layer-by-layer assembly techniques (Tang et al., 2006). For example, Matsusaki et al. (2007) fabricated a multilayer fibroblast construct by preparing fibronectin–gelatin nanofilms. In a similar way, Kawecki et al. (2018) applied human osseous cell sheet as living biopaper to support laser-assisted bioprinting of human endothelial cells.

**Figure 4:**
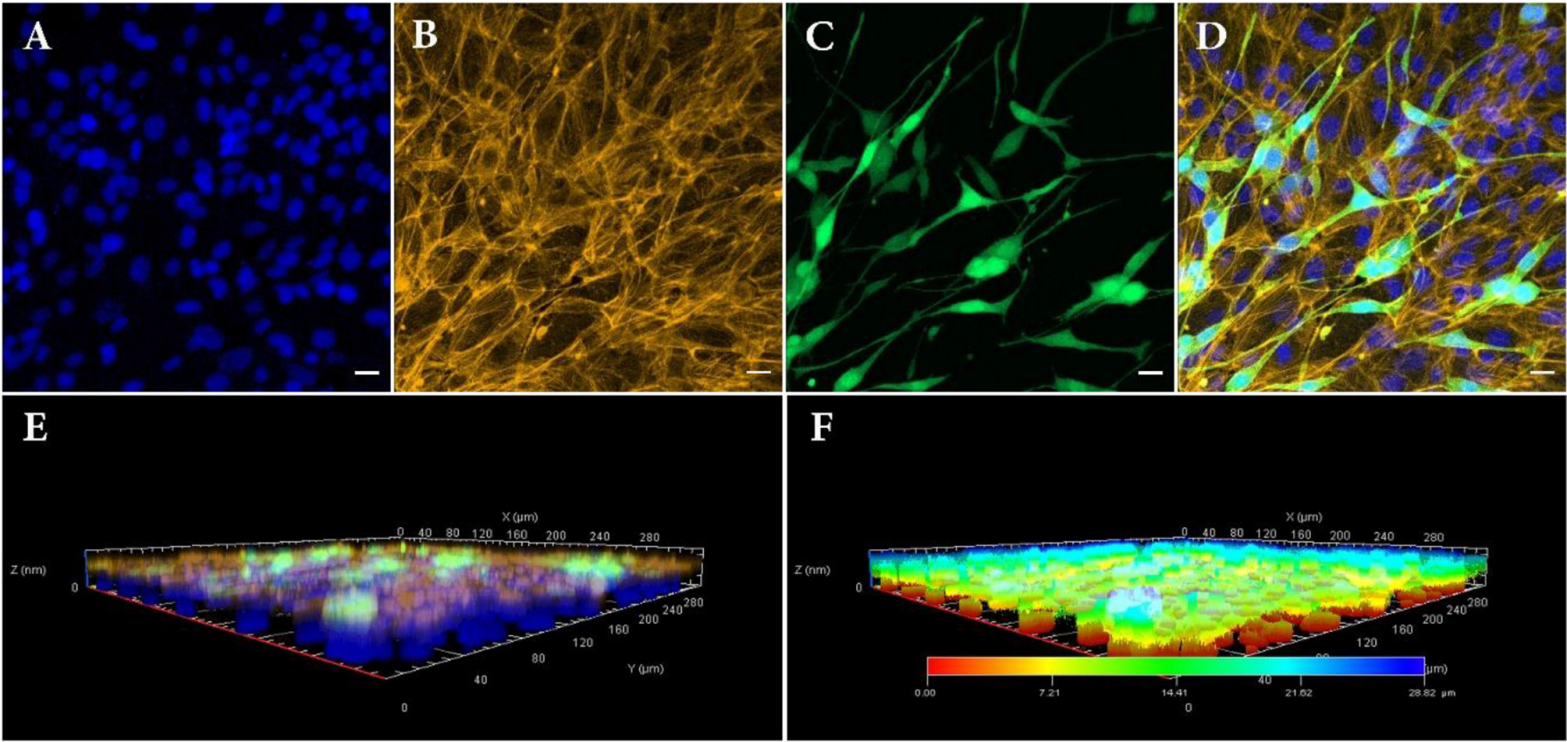
Confocal fluorescence images of bilayer bioprinted construct. **(A)** Counterstained nuclei with DAPI (blue). **(B)** Actin filament staining with phalloidin (Yellow). **(C)** GFP positive NIH3T3 cells (Green). **(D)** Merged image. **(E)** 3D view and **(F)** depth coding of bilayer bioprinted construct. All images were captured on day 3 after printing of NIH3T3 or day 10 after printing RPE cells. Scale bars: 100 μm.

RPE cells were printed with density of 110 ± 15 RPE cells per deposition, calculated according to their concentration as well as their size. One week after bioprinting, RPE cells formed a monolayer sheet, ready to be used as living biopaper for bioprinting NIH3T3 cells. Figures 4A–D depict the distribution of the two cell types 3 days after printing the second layer (i.e., 10 days after printing the first layer). As NIH3T3 cells are constitutively producing GFP, they are easily distinguished from RPE cells, especially in merged images (Figure 4D).

As a matter of fact, RPE cells were homogeneously distributed as a dense layer over the GelMa surface whereas NIH3T3 cells randomly positioned themselves in a second and less dense layer (160 depositions against 324 depositions for RPE). Separate layers organization of printed cells was also confirmed with 3D confocal imaging (Figures 4E,F). Indeed, these images bring evidences that the RPE unlabeled cells (and their nuclei) have been localized in the lowest part of the construct, while GFP positive NIH3T3 cells can be found only on its upper layer. Moreover, depth coding of samples revealed that the full thickness of the construct is around 28.82 μm, consistent with cells' size (Figure 4F). Although size, shape and structure of RPE cells depend on age and location, average thickness of a RPE sheet in human is about 14 μm (Forrester et al., 2016). Similarly, NIH3T3 cells size is estimated to be around 15 μm, leading to a theoretical 2-layer construct of 29 μm.

Overall, the capability to direct-write cell using a bioprinting strategy is not only a first step toward successful multilayer printing of dense cells but also a critical indicator of the feasibility of the envisioned organ printing technology. In the absence of a hydrogel ink, we can list the following impacts for the direct-write bioprinting method particularly from the viewpoint of cellular function and tissue remodeling.

First, as the most important point, cell-cell crosstalk is well-established, and the activation of notch signaling for regulating communication between neighboring cells can be guaranteed.

Second, bioprinting is performed with a high density of cells, which is particularly important for generating cell-rich tissue models. This breakthrough is directly dependent on the cell density within bioprinted construct. Indeed, different cells population behaviors, such as development capability and expression of differentiation factors are inefficient when only a small number of cells are present (Payne and You, 2014).

Finally, the ability to create heterogeneous layered models from different cell sources is another advantage of using cell suspensions as printing ink. Obviously, in hydrogel-based bioprinting systems, bioink properties, such as material type, surface tension and viscosity, shall be set according to the cell sources. Furthermore, shear tensions originated from high viscous hydrogels, may adversely affect cell viability.

Here we selected RPE and NIH3T3 cells just for proof-of-concept study, and it is important that in the future, cells' type are being chosen according to the ultimate goal in a more physiologically relevant arrangement. In such case cell-cell communications can assure the transmission of vital molecular signals and trigger cell differentiation and remodeling. Future work by this approach may also look into successive printing of more than two cell layers.

## Conclusion

It is now clear that bioprinting is a powerful technique with many potential applications for localizing biological components into 3D-engineered structures. Considerable progresses have been made and described in literatures to design and synthesize various hydrogel-based bioinks, compatible with living cells and their microenvironment. These models usually lack uniformity of printed cells and thereupon cannot completely establish immediate vital cellular communications for survival and remodeling of multicellular complex tissues. Another challenge related to application of hydrogels as bioink is that high viscosity of applied materials may generate high shear stress during cell deposition. Therefore, in the current study, we successfully replaced hydrogel ink with cell suspension and set size and distances between inkjet depositions. In this regards, not only no clogging occurred within the capillary nozzle, but also, the calculated maximum shear stress was relatively low. Moreover, thanks to the low viscosity of the cell suspension, we were able to print cells at high density (e.g., 110 ± 15 RPE cells per deposition). After adjusting density and uniformity of printed cells, current hydrogel-free bioprinting approach was extended to directly write two cellular layers on top of each other. To do so, we had first bioprinted a homogeneous epithelium layer, and once the cells covered all the surface, a second layer of cells was printed following first deposition pattern. Here we just applied two different cell sources (NIH3T3 and RPE cells) as models, but for clinical applications, cells must be targeted according to the ultimate goal. To sum up, such direct-write bioprinting strategy is a considerable step forward to the successful printing of complex multicellular tissues, where high density cell layers communicate with each other through direct contact.

## Data Availability Statement

All datasets generated for this study are included in the article/supplementary material.

## Author Contributions

EM and CM conceived and designed the experiments, performed the experiments, analyzed the data, and wrote the paper.

### Conflict of Interest

The authors declare that the research was conducted in the absence of any commercial or financial relationships that could be construed as a potential conflict of interest.
